# A scoping review on the mental health of disaster responders for natural disasters in Japan

**DOI:** 10.1002/pcn5.70346

**Published:** 2026-05-22

**Authors:** Yukari Ito, Sho Takahashi, Hirokazu Tachikawa, Haruhiko Midorikawa, Tetsuaki Arai

**Affiliations:** ^1^ Doctoral Programme in Medical Sciences, Graduate School of Comprehensive Human Sciences University of Tsukuba Ibaraki Japan; ^2^ Department of Disaster and Community Psychiatry, Institute of Medicine University of Tsukuba Tsukuba Ibaraki Japan; ^3^ Department of Psychiatry, Institute of Medicine University of Tsukuba Tsukuba Ibaraki Japan; ^4^ Department of Psychiatry, Division of Clinical Medicine, Institute of Medicine University of Tsukuba Tsukuba Ibaraki Japan

**Keywords:** disaster responders, mental health, natural disasters, post‐traumatic stress, psychological distress

## Abstract

This study addressed a significant gap in the literature on disaster responders’ mental health. Numerous studies have examined disaster victims’ mental health. However, reviews on the mental health of disaster responders are limited. Thus, this study reviewed the existing literature and clarified the mental health effects according to disaster type and occupation in Japan. The PubMed, CiNii, Web of Science, Google Scholar, and PsycInfo databases were searched. The inclusion criteria were natural disasters in Japan and reports on the mental health status of disaster support workers. The search period was from 1997 to 2025. Among 664 studies, 55 met the inclusion criteria. The research methods included 39 cross‐sectional and 16 longitudinal studies. Various types of disasters were identified. The main reported mental health symptoms were post‐traumatic stress state (PTSS) and psychological distress. The prevalence of and factors contributing to worsening PTSS and psychological distress varied across occupations. PTSS and psychological distress were relatively high among medical personnel, local government employees, and nuclear power plant workers and relatively low among firefighters and Japan Ground Self‐Defense Force personnel. Several personal, disaster‐related, and occupational factors were identified as exacerbating PTSS and psychological distress. These included home damage, experiences of discrimination and abuse, long working hours, lack of rest, and poor workplace communication. The prevalence rates and identified stressors should be used to enhance training and preparedness measures during peacetime.

## Graphical abstract

This scoping review synthesized 55 studies (1997–2025) on the mental health of disaster responders in Japan following natural disasters. Mental health outcomes varied by occupation, with higher prevalence among healthcare workers, local government employees, and nuclear power plant workers, and lower prevalence among firefighters and JGSDF personnel. Key risk factors included home damage, discrimination, long working hours, and poor workplace communication. These findings highlight the importance of strengthening preparedness, training, and mental health support systems during peacetime.

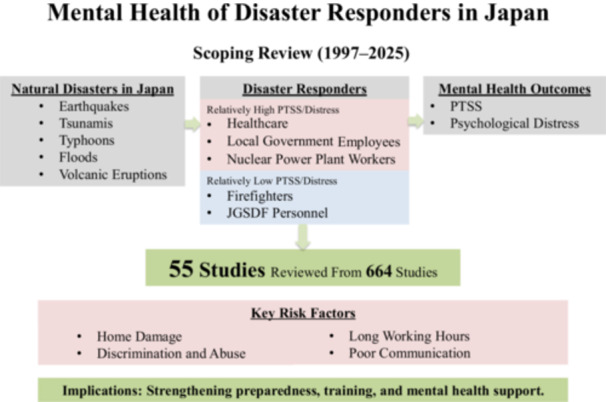

## INTRODUCTION

Japan has experienced frequent natural disasters, including earthquakes, tsunamis, typhoons, floods, forest fires, and snowstorms. In affected areas, emergency service personnel and public sector workers, such as firefighters, emergency medical technicians, police officers, Japan Ground Self‐Defense Force (JGSDF) members, local officials, and infrastructure employees, engage in rescue and relief operations. These disaster relief workers^1^ are responsible not only for evacuations, searches, and body recovery but also for long‐term tasks such as radiation decontamination. Some are dispatched from outside the affected areas, whereas others are local residents who are also victims, facing the dual burden of personal loss and professional duties. Such occupational stressors can cause mental health problems; therefore, their mitigation is essential.^2^

The Great Hanshin‐Awaji Earthquake (1995) drew significant attention to disaster‐related mental health.^3^ While many victims recover, some develop post‐traumatic stress disorder (PTSD).^4^ The Great East Japan Earthquake (GEJE, 2011), which encompassed an earthquake, tsunami, and nuclear accident, caused widespread psychological distress, PTSD, depression, anxiety, and sleep disorders.^5^, ^6^ Reported risk factors include gender, unemployment, economic hardship,^7^, ^8^ lack of support from family or neighbors,^9^ men under 65 years living alone,^10^ alcohol consumption,^11^ and pre‐existing mental illness.^12^

Following the GEJE, research has increasingly examined the mental health of both relief workers and victims.^5^ However, most studies have focused on specific occupations, and few have comprehensively analyzed mental health problems across disasters and job types. Moreover, the literature is methodologically heterogeneous with respect to study design, target populations, and outcome measures, limiting the feasibility of quantitative synthesis.

Japan represents a particularly informative context for such a review because of the frequency and diversity of natural disasters, which have generated a substantial body of research on disaster responders. Furthermore, disaster relief workers in Japan operate within a distinctive institutional and cultural framework characterized by unique public service systems and occupational structures. Focusing on Japan therefore enables an in‐depth exploration of occupational and contextual factors influencing the mental health of disaster relief workers. Accordingly, this study aimed to identify factors contributing to the deterioration of mental health among disaster relief workers by examining personal characteristics, disaster exposure, and work‐related conditions and to systematically organize existing knowledge through a scoping review of natural disasters in Japan. A scoping review was selected because the available literature on the mental health of disaster responders in Japan is heterogeneous in terms of study design, populations, and outcomes, precluding quantitative synthesis. In addition, whereas existing systematic reviews have primarily focused on disaster victims, this review specifically maps the scope and characteristics of research on occupational groups involved in disaster response.

## METHODS

This review followed the framework of Arksey and O'Malley^13^ and the Preferred Reporting Items for Systematic Reviews and Meta‐Analyses extension for Scoping Reviews (PRISMA‐ScR).^14^

### Search strategy

A comprehensive search was conducted in five databases—PubMed, CiNii, Web of Science, Google Scholar, and PsycInfo—on July 12, 2025. Only articles published in Japanese or English were included. The search terms included *Japan*, *disaster*, *mental health/stress*, and occupations such as health personnel, government employees, emergency responders, firefighters, and police. The detailed search queries for each database are listed in Table 1. The search period spanned 1997–2025, covering major events including the Great Hanshin‐Awaji Earthquake and the GEJE.

**Table 1:** Detailed search queries for each database.

Database	Search queries
PubMed	Japan AND (“Disasters”[Mesh] OR disaster) AND (“Mental Health”[Mesh] OR mental health) AND (“Stress, Psychological”[Mesh] OR stress) AND (worker OR employer OR emergency responder OR firefighter OR police OR government employer OR health personnel)
CiNii	(Disaster OR 被災 [Japanese]) AND (mental health OR stress) AND (職 [Japanese] OR worker OR employer OR emergency responder OR firefighter OR police OR government employer OR health personnel)
Web of Science	Japan AND disaster AND (mental health OR stress) AND (worker OR employer OR emergency responder OR firefighter OR police OR government employer OR health personnel)
Google Scholar	(allintitle: Japanese disaster mental health worker) OR (allintitle: employer) OR (allintitle: emergency) OR (allintitle: responder) OR (allintitle: firefighter) OR (allintitle: police) OR (allintitle: government) OR (allintitle: health) OR (allintitle: personnel)
PsycInfo	Japan AND disaster AND (mental health OR stress) AND (worker OR employer OR emergency responder OR firefighter OR police OR government employer OR health personnel)

### Inclusion and exclusion criteria

Eligible studies focused on natural disasters in Japan (e.g., earthquakes, typhoons, floods, tsunamis, and volcanic eruptions) and examined the mental health of disaster relief workers. Reviews, commentaries, editorials, conference abstracts, case reports, activity reports, intervention studies, qualitative studies, and articles in languages other than Japanese or English were excluded.

### Data charting, analysis, and synthesis of results

After the comprehensive search, all records were exported to EndNote^**®**^ (version 21.5; Clarivate), where duplicates were removed automatically and manually. Titles and abstracts were screened, followed by full‐text review to determine eligibility. Four psychiatrists independently evaluated the papers and resolved discrepancies through consensus. The extracted attributes included authors, year, paper type, country, disaster type and year, target occupation, survey period, mental health outcomes, and factors contributing to deterioration related to personal attributes, disaster conditions, and job content.

### Ethical considerations

All studies were summarized without altering their original intent, and the sources were fully cited.

### Use of artificial intelligence tools

The original Japanese manuscript was translated into English with the assistance of ChatGPT (OpenAI; accessed February 2026). The authors reviewed and edited the output to ensure accuracy. The AI tool was used solely for language editing and did not contribute to the study design, data analysis, or interpretation. The authors take full responsibility for the content of this manuscript.

## RESULTS

**Figure 1:**
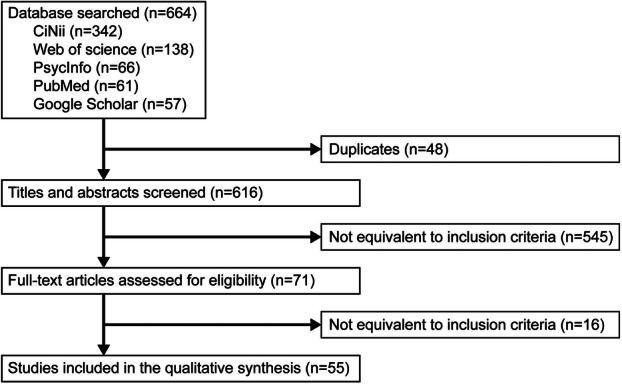
The screening procedure.

In total, 664 records were identified from PubMed (61), CiNii (342), Web of Science (138), Google Scholar (57), and PsycInfo (66). After removing 48 duplicates using EndNote^**®**^ and manual review, 616 records remained. After screening titles and abstracts, 545 studies were excluded, leaving 71 studies for full‐text review. Subsequently, six reviews, one activity report, one intervention study, one qualitative study, and seven ineligible documents were excluded. Ultimately, 55 studies met the inclusion criteria (Figure 1).

Table 2 summarizes the 55 studies—18 in Japanese and 37 in English—all of which were published after 2011. Thirty‐nine were cross‐sectional and 16 were longitudinal, with survey periods ranging from immediately after the disaster to 7 years later. Most addressed earthquakes (*n* = 51), with a few addressing floods (*n* = 1), volcanic eruptions (*n* = 1), and multiple disasters (*n* = 2).

**Table 2:** Summary of findings from the scoping review.

Author(s)	Timing	Participants	Assessment tool	Cutoff points	Prevalence (%)	Main findings
Healthcare workers						
Sakuma et al.^15^	14, 30, 43, 54 months after the GEJE	Local medical workers	PCL‐S	≥44	8.3 4.6 4.8 5.3	Disaster work, poor communication, lack of rest, relocation, family loss, and near‐death experiences are linked to persistent severe and fluctuating PTSS.
Nishi et al.^16^	1, 4 months after the GEJE	Members of disaster medical assistance teams	PDI IES‐R	–	–	PDI at 1 month predicts IES‐R at 4 months. ≥4 h/day of TV viewing at 1 month predicts PTSS at 4 months.
Sato et al.^17^	1 year after the GEJE	Nurses from a general hospital located within 50 km of the Fukushima Daiichi Nuclear Power Plant	IES‐R K6 K6 K6 RS‐14	≥25 ≥13 ≥10 ≥5 –	26.3 11.9 24.6 55.9 –	Evacuation and difficulty obtaining leave are associated with PTSS. Supervisor support and resilience are negatively correlated with anxiety and depression.
Tominaga et al.^18^	5 months after the GEJE	Clinical psychologists dispatched to schools in Miyagi, Iwate, and Fukushima	IES‐R PTG ProQOL‐5	– – –	– – –	Child death and disappearance are associated with PTSS. Intrusion severity is the strongest predictor of PTG. Women and those who received professional support have higher PTG. Prior knowledge increases compassion satisfaction and reduces burnout.
Tsutsui et al.^19^	8 months after the GEJE	Employees of a public general hospital	IES‐R CES‐D ICG	≥25 ≥16 ≥26	29.3 37.8 9.8	Prolonged grief differs qualitatively from PTSS and distress. Grief symptoms are independent of PTSS and depression.
Kawashima et al.^20^	1 month and 4 years after the GEJE	Members of Disaster Medical Assistance Teams	IES‐R PDI MBI	– – –	– – –	PDI at 1 month predicts IES‐R and MBI at 4 years.
Shoji et al.^21^	4 and 12 months after the GEJE	Local medical workers	IES‐R	≥25	43.8 (4 months) 44.4 (12 months)	Nursing, older age, long job tenure, bereavement, home loss, work stress, long working hours, health problems, and tsunami anxiety are linked to PTSS. Restoration of essential services reduces PTSS.
Kawashima et al.^22^	2 years after the GEJE	Staff working at facilities for individuals with intellectual disabilities	IES‐R GHQ‐12	≥25 ≥4	25.3 54.4	Staff shortages and deteriorating business conditions are related to PTSS. No significant predictors emerged in GHQ‐12 regression analysis.
Matsuoka et al.^23^	1 month after the GEJE	Members of Disaster Medical Assistance Teams	K6 CES‐D PDI IES‐R	≥13 ≥16 ≥23 ≥25	4.0 21.4 – –	Radiation concerns are strongly linked to psychological distress.
Nukui et al.^24^	4 years after the GEJE	Nurses working at medical institutions in Fukushima Prefecture	GHQ‐12	≥4	45.6	Radiation fear, work–life burden, and lack of a spouse are associated with psychological distress.
Yamazaki et al.^25^	4 years after the GEJE	Local hospital nurses (management positions)	GHQ‐12	≥4	50.0	Small hospitals, poor workplace relationships, and conflict are linked to intention to resign.
Hirano et al.^26^	4 years after the GEJE	Nurses dispatched to the GEJE	GHQ‐12 Coping Scale^27^	– –	– –	Mental wellness is correlated with stress experiences and systematic stress management measures.
Hatakenaka et al.^28^	Just before departure; during activities; immediately after activities; 1, 2, 3, and 4 weeks after activities	Members of Disaster Psychiatric Assistance Team	Compassion Fatigue/Satisfaction Scale	–	–	Compassion fatigue followed two patterns: early increase, or decrease during/after activity followed by later increase.
Matsukiyo et al.^29^	3 years after the GEJE	Nurses of Disaster Medical Assistance Teams	IES‐R	≥25	7.1	Cognitive stress appraisal and subsequent negative emotions affect long‐term maladjustment. In women, uncontrollable situations with problem‐focused coping and humor reduce maladjustment. In men, life‐saving activity and negative self‐evaluation increase maladjustment.
Yonemoto et al.^30^	3 years after the GEJE	Local medical workers	–	–	–	29% of doctors and 64.5% of nurses intended to resign or change careers.
Local government employees						
Maeda et al.^31^	2 years after the GEJE	Local municipality workers (within 30 km of a nuclear power plant)	IES‐R MINI	≥25 –	21.6 17.9	Residents’ negative emotions and anger are linked to PTSS and depression.
Takahashi et al.^32^	4 years after the 2016 Kumamoto Earthquake; 2 years after the 2018 West Japan heavy rain	Local municipality workers	IES‐R K6	≥25 ≥10	8.6 6.0	Workplace conflicts, heavy workload, residents’ trauma, and poor self‐control are linked to PTSS. Conflicts, workload, and residents’ trauma are associated with mood and anxiety disorders.
Ueda et al.^33^	20–22 months after the GEJE	Workers of local SHAKYOs (local social welfare workers) in tsunami‐affected areas	PCL‐S PHQ‐9 K6	≥44 ≥10 ≥13	4.0 12.3 7.9	Resident criticism, poor workplace communication, and relocation are linked to PTSS. Near‐death experiences, colleague loss, criticism, relocation, and poor communication are linked to depression and distress.
Okano et al.^34^	6 months after the 2016 Kumamoto Earthquake	Kumamoto municipality workers	IES‐R	≥25	14.9	Female sex, non‐management positions, lack of workplace communication, and lack of family time are associated with PTSS.
Tsuno et al.^35^	6 months after the GEJE	Local municipality workers in the Kanto region affected by liquefaction	IES‐R CD‐RISC	≥25 –	20.1 –	Personal disaster experience, injuries, home damage, and low resilience are associated with PTSS. High resilience was observed in ≤25‐year‐olds without chronic disease.
Takahashi et al.^36^	1 year after the GEJE	Miyagi municipality workers	PTSS Prevention Checklist^37^	–	–	Female sex and coastal areas are associated with PTSS.
Fukasawa et al.^38^	2, 7, 16 months after the GEJE	All public servants in the Miyagi prefectural government	K6	≥10	9.6 (2 M) 9.5 (7 M) 9.3 (16 M)	Living away from home is strongly linked to psychological distress.
Suzuki et al.^39^	2, 7 months after the GEJE	All public servants in the Miyagi prefectural government	K6	≥10	9.6 (7 M)	Poor workplace communication, lack of weekly rest, resident complaints, bereavement, and >2 months in shelters increased risk of psychological distress.
Wakashima et al.^40^	3, 7, 15, 23 months after the GEJE	Local government workers	K6 CSI^112^	– –	– –	13.55% had high stress 15 months post‐earthquake; home damage and displacement were linked to stress.
Suzuki et al.^41^	16 months after the GEJE	Local government workers	MBI‐GS	Exhaustion ≥ 75th percentile + (Cynicism ≥ 75th percentile or Professional efficacy ≤ 25th percentile)	15.9	Home destruction, health/welfare duties, ≥80 h overtime work, poor workplace communication, shelter life, and resident complaints are linked to burnout.
Yoshida et al.^42^	4 years after the GEJE	Public health nurses in Fukushima Prefecture	SOC‐13	–	–	Age <40 years, <10 years of experience, and staff nurse position were associated with high anxiety.
Yamada et al.^43^	1–2 months after the GEJE (during assignment) 4–5 months after the GEJE (3 months after being dispatched)	Public health nurses dispatched to disaster areas	Brief Job Stress Questionnaire	Psychological stress ≥ 51 or physical stress ≥ 27	11.5	Anxiety, sadness, and insomnia increased during activity. Anger, irritability, and poor concentration increased 3 months post‐dispatch.
Yamada et al.^44^	1 year after the GEJE	Local government workers in Iwate, Miyagi, and Fukushima Prefectures	Self‐reported stress symptoms^45^, ^46^	–	21.3 (Fukushima 26.3 Miyagi 18.9 Iwate 13.3)	Fukushima and Miyagi residence, age <30 years, illness treatment, home loss, fewer holidays, poor preparedness, lack of counseling, radiation concerns, corpse handling, and residents’ abuse are associated with stress responses.
Takahashi et al.^47^	1 year after the GEJE	Miyagi municipality workers	IES‐R	≥25	50.4	A 17‐item Acute Stress Response Scale^36^ was developed to measure acute stress in local officials within 1 month post‐disaster, with defined cutoff values.
Employees and subcontractors at the Fukushima Daiichi and Fukushima Daini Nuclear Power Plants						
Ikeda et al.^48^	1, 14–15, 32, 44 months after the GEJE	Full‐time TEPCO employees of the Fukushima Daiichi and nearby Daini Nuclear Power Plants	IES‐R K6	≥25 ≥13	26.3 13.1	Near‐death experiences, property loss, discrimination/abuse, and tsunami evacuation are linked to PTSS and distress; discrimination/abuse had prolonged effects.
Ikeda et al.^49^	2–3 months and 1, 2, 3 years after the GEJE	Full‐time TEPCO employees of the Fukushima Daiichi and nearby Daini Nuclear Power Plants	AIS IES‐R	≥6 ≥25	56.2 (2–3M) 39.9 (1Y) 46.6 (2Y) 46.9 (3Y) 26.6 (2–3M)	Discrimination/abuse, tsunami evacuation, explosions, livelihood loss, property loss, and evacuation life are linked to insomnia; discrimination/abuse had lasting effects.
Shigemura et al.^50^	2–3 months after the GEJE	Full‐time TEPCO employees of the Fukushima Daiichi and nearby Daini Nuclear Power Plants	IES‐R PDI	≥25 ≥23	– –	PDI, discrimination, and pre‐existing illnesses are associated with PTSS.
Tajima et al.^51^	2–3, 14‐15, 32, 44 months after the GEJE	Full‐time TEPCO employees of the Fukushima Daiichi Nuclear Power Plant	IES‐R K6	≥25 ≥13	30.6 15.8	Field engineers engaged in prolonged nuclear plant restoration work are linked to PTSS and distress.
Tanisho et al.^52^	2–3 and 14–15 months after the GEJE	Full‐time TEPCO employees of the Fukushima Daiichi and nearby Daini Nuclear Power Plants	IES‐R K6 PDI	– – –	– – –	PTSS at 14–15 months was predicted by early PTSS, older age, and discrimination/abuse. Distress at 14–15 months was predicted by early distress and discrimination/abuse.
Nagaoka et al.^53^	4–12 months after the GEJE	Radiation control and non‐destructive inspectors in nuclear plant inspections	GHQ‐12 GHQ‐12 TAC‐24^54^	≥3 ≥4 –	49.0 36.5 –	Post‐accident inspectors using avoidance or resignation coping had higher distress.
Takahashi et al.^55^	2–3 months after the GEJE	Full‐time TEPCO employees of the Fukushima Daiichi and nearby Daini Nuclear Power Plants	IES‐R	≥25	–	Age 20–40 years, managerial role, discrimination, and abuse are linked to workplace interpersonal support (WIS). WIS was not significantly associated with PTSS.
Okazaki et al.^56^	3 years after the GEJE	Full‐time TEPCO employees of the Fukushima Daiichi Nuclear Power Plant	–	–	–	Radiation education was associated with reduced anxiety.
Hidaka et al.^57^	6 years after the GEJE	Operation leaders of radioactivity decontamination workers in Fukushima	–	–	–	Work environment knowledge was linked to radiation anxiety.
Hidaka et al.^58^	2 years after the GEJE	Radioactivity decontamination workers in Fukushima	–	–	–	Isolation, lack of a written contract, and older age were linked to radiation anxiety.
JGSDF personnel						
Dobashi et al.^59^	3 months after the GEJE (1 month after deployment)	JGSDF personnel dispatched to the GEJE	IES‐R K10	≥4 (median split) ≥25	– 3.0	IES‐R scores were low (the mean [±SD] = 6.2 ± 8.1). Exposure to dead bodies and younger age were significant IES‐R factors. K10 scores were low (mean [±SD] = 12.8 ± 4.4); younger age was a significant factor.
Nagamine et al.^60^	1, 6 months, and 1 year post‐mission completion	JGSDF personnel dispatched to the GEJE	IES‐R K10	≥25 ≥25	3.7 0.8 0.7 2.2 0.5 0.5	Shelter‐site camping was a significant risk factor for high PTSS and distress.
Nagamine et al.^61^	1, 6 months and 1, 2, 3, 4, 5, 6 years post‐mission completion	JGSDF personnel dispatched to the GEJE	IES‐R	≥25	2.7 1.7 1.1 1.4 0.9 0.9 1.5 1.0	Personal disaster experience, ≥3‐month deployment, older age, and ≥3‐month post‐deployment overtime were linked to PTSS.
Saito et al.^62^	1, 6 months and 1, 2, 3, 4, 5, 6 years post‐mission completion	JGSDF personnel dispatched to the GEJE	IES‐R	–	–	A minority developed late‐onset or chronic PTSS. Risk factors included older age, personal disaster experience, body recovery, radiation risk, longer deployment, lack of timely leave, and prolonged overtime.
Firefighters						
Fushimi et al.^63^	Shortly, 2 weeks, and 1 month after return from rescue efforts	Male firefighters dispatched to the GEJE from a disaster‐unaffected area (Akita City)	IES‐R	≥25	1.7 (shortly) 0.0 (2 W) 0.0 (1 M)	Few or no participants showed PTSS.
Noda et al.^64^	Within 6 months of the GEJE	Firefighters or emergency medical technicians dispatched to the GEJE	IES‐R CD‐RISC	≥24 –	25.0 –	Higher education and resilience reduced PTSS.
Nojima et al.^65^	5 months after the GEJE	Firefighters dispatched from other regions to GEJE‐affected areas	IES‐R	≥25	0.0	No participants showed PTSS.
Yoo et al.^66^	1.5 years after deployment to the GEJE	Firefighters dispatched to the disaster area	IES‐R K6	≥25 ≥10	3.6 0.9	Older age was associated with higher PTSS. Participants showed both PTG and PTSS. Colleague and family support promoted PTG. Acute stress predicted depression 1.5 years later.
Police officers						
Kamijo et al.^67^	3 months after the Mt. Ontake eruption	Local police officers	PDS CD‐RISC	Mild 1 – 10 Moderate 11 –20 Moderate to severe 21 –35 Severe 36~ High 62– Medium 50 – 61 Low 1 – 49	Mild 25.8 Moderate 0.9 Severe 0.0 –	>7 workdays, coping through drinking or smoking, female sex, and support for victims’ families were linked to greater PTSS severity.
Preschool teachers						
Sasaki et al.^68^	1 month after the GEJE	Preschool teachers in Fukushima	CES‐D Preschool Teacher Efficacy Scale 10‐item version^69^	≥16 –	39.7 –	Preschool teacher efficacy was protective against depression.
Studies involving multiple professions						
Sakuma et al.^70^	14 months after the GEJE	Local firefighters Local municipality workers Local medical workers	PCL‐S PHQ‐9 K6	≥44 ≥10 ≥13	2.6 9.0 9.3 5.6 24.4 22.0 2.6 14.9 14.5	Municipal and medical workers had higher PTSS and depression than firefighters. Lack of rest and poor communication, as well as disaster‐related work, increased PTSS, depression, and distress.
Usami et al.^71^	7 months and 1 year after the GEJE	Local firefighters; Special Rescue Team of the Japan Coast Guard; rescue divers	IES‐R	≥25	28.8 6.3 20.0 13.5 0.0 0.0	Casual conversation about the trauma had no effect on PTSS.
Yamazaki et al.^72^	Part 1: 1 month after the 2007 Niigata‐ken Chuetsu‐oki Earthquake Part 2: 22 months after the 2004 Niigata‐ken Chuetsu Earthquake	Part 1: Caregivers working at aged care centers or facilities for individuals with mental illness Part 2: Local nurses	Part 1: IES‐R GHQ‐12 Part 2: IES‐R	≥25 ≥4 ≥25	Part 1: 19.5 52.1 Part 2: 7.9	Part 1: Lack of water and food, household belongings, safe beds, and aftershocks were associated with PTSS; home destruction worsened GHQ‐12. Part 2: Older age was associated with higher levels of re‐experiencing/intrusion and avoidance symptoms.
Kobayashi et al.^73^	5 years after the 2004 Niigata‐Fukushima heavy rain and the 2004 Niigata‐ken Chuetsu Earthquake; 2 years after the 2007 Niigata‐ken Chuetsu‐oki Earthquake	Local hospital nurses and public health nurses	IES‐R	≥25	7.0	Personal disaster experience, home destruction, female sex, poor personal or family health, and shelter life were linked to PTSS.
Abe et al.^74^	2 years after Typhoon No.12 (2011)	Local government officers and firefighters in Wakayama Prefecture	–	–	–	Lack of information to respond to residents’ inquiries was more stressful than residents’ complaints.
Hirano et al.^75^	3–4 years after the 2004 Niigata Chuetsu Earthquake	Public health nurses, hospital nurses, and emergency personnel in Niigata Prefecture	53‐item stressful events questionnaire	–	–	Female sex, hospital/health center employment (vs. fire departments), and residential relocation due to the disaster were associated with higher stress.

*Note*: A dash (–) indicates that the assessment tool, cutoff values, or prevalence were not reported or not applicable.

Abbreviations: AIS, Athens Insomnia Scale; ASR, Acute Stress Response; CD‐RISC, Connor–Davidson Resilience Scale; CES‐D, Center for Epidemiologic Studies Depression Scale; CSI, Comprehensive Stress Response Inventory; GEJE, Great East Japan Earthquake; ICG, Inventory of Complicated Grief; IES‐R, Impact of Event Scale‐Revised; JGSDF, Japan Ground Self‐Defense Force; K6/K10, Kessler Psychological Distress Scale (6‐item and 10‐item versions); MBI, Maslach Burnout Inventory; MBI‐GS, Maslach Burnout Inventory–General Survey; MINI, Mini‐International Neuropsychiatric Interview; PCL‐S, Post‐Traumatic Stress Disorder Checklist‐Screening Version; PDI, Peritraumatic Distress Inventory; PHQ‐9, Patient Health Questionnaire‐9; ProQOL‐5, Professional Quality of Life Scale‐5; PTG, Post‐traumatic growth; PTSS, Post‐traumatic stress state; RS‐14, Resilience Scale Short Version; SOC‐13, Sense of Coherence–13; TAC‐24, Tri‐axial Coping Scale; TEPCO, Tokyo Electric Power Company Holdings; Incorporated; WIS, workplace interpersonal support.

In total, 48 studies addressed the GEJE (2011); the remainder examined the Kumamoto (2016), Niigata Chuetsu (2004), and Chuetsu‐oki (2007) earthquakes. Floods were reported in the Niigata‐Fukushima (2004) and Western Japan (2018) events, and volcanic eruptions were noted in the Mount Ontake eruption (2015). Regarding occupations, 15 studies involved medical professionals, 14 local government staff, 10 nuclear plant employees, four JGSDF members, four firefighters, one police officer, one kindergarten teacher, and six studies involved multiple occupations.

The healthcare workforce comprised hospital doctors, nurses, other hospital staff, members of Disaster Medical Assistance Teams (DMAT) and Disaster Psychiatric Assistance Teams (DPAT), and care workers in nursing and disability facilities. Studies involving multiple professions included local government employees, firefighters, and coast guard officers.

The mental health outcomes measured were post‐traumatic stress state (PTSS) (*n* = 36), psychological distress (*n* = 21), burnout (*n* = 2), occupational stress (*n* = 1), grief (*n* = 1), insomnia (*n* = 1), and compassion fatigue (*n* = 1). The most common PTSS measure was the impact of event scale‐revised (IES‐R),^76^ followed by the peritraumatic distress inventory (PDI)^77^ and the PTSD checklist‐screening version (PCL‐S),^78^ each used in a limited number of studies. Psychological distress was assessed using a range of instruments and classified according to the type of measurement. Measures of general mental health included the Kessler Psychological Distress Scale (K6, K10)^79^ and the 12‐item General Health Questionnaire (GHQ‐12).^80^ Depression‐related outcomes were evaluated using the Center for Epidemiologic Studies Depression Scale (CES‐D)^81^ and the Patient Health Questionnaire‐9 (PHQ‐9).^82^ Diagnostic assessments were conducted using the Mini‐International Neuropsychiatric Interview (MINI).^83^ Burnout syndrome was evaluated using the Maslach Burnout Inventory‐General Survey (MBI),^84^, ^85^ occupational stress using the Occupational Stress Questionnaire,^86^ grief reactions using the Inventory of Complicated Grief (ICG),^87^ insomnia using the Athens Insomnia Scale (AIS),^88^ and compassion fatigue/satisfaction using the Compassion Fatigue/Satisfaction Scale.^89^, ^90^ Outcomes related to protective factors included resilience (*n* = 4), coping (*n* = 2), and childcare efficacy (*n* = 1). The assessment scales were the Resilience Scale Short Version (RS14),^91^ the Connor–Davidson Resilience Scale (CD‐RISC),^92^ the Tri‐axial Coping Scale (TAC‐24),^54^ and the 10‐item Caregiver Efficacy Scale.^69^

Table 3 presents the factors associated with worsening PTSS, and Table 4 presents the factors associated with psychological distress by job type.

**Table 3:** Factors contributing to the worsening of PTSS by job type.

Occupation	Personal factors	Disaster factors	Business factors
Common to all occupations		Damage to home Near‐death experience Death or disappearance of family members	Long working hours Insufficient rest Lack of communication in the workplace
Healthcare workers	Old age Negative self‐evaluation Emotion‐focused coping Anxiety about future life	Death or disappearance of family members or friends Near‐death experience Total destruction of home Evacuation Relocation	Insufficient rest Vacation time Poor workplace communication Nurses Human resources and management department managers Stressful work schedule
Local government employees	Female Lack of time with family Non‐managerial positions	Death or disappearance of family members Near‐death experience Injuries Damage to home Relocation	Poor workplace communication Complaints, anger, and criticism from residents Insufficient rest Disaster‐related work Job reassignment Lack of autonomy in work
Employees and subcontractors at the Fukushima Daiichi and Fukushima Daini Nuclear Power Plants		Evacuation from tsunami Near‐death experience Significant loss of property	Discrimination and harassment Work immediately after the disaster On‐site restoration work
Ground self‐defense force personnel	40 years old or older	Being a disaster victim	Three months or more of temporary work Long working hours after temporary work Body recovery work Work involving radiation exposure risk Living on the same premises as disaster victims
Firefighters	Single 50 years old or older		Early arrival at disaster sites Ambulance workers Three or more dispatches
Police officers	Female drinks alcohol and smokes		Dispatches lasting seven days or longer Support for families of disaster victims

Abbreviation: PTSS, post‐traumatic stress state.

**Table 4:** Factors contributing to the worsening of psychological distress by job type.

Occupation	Personal factors	Disaster factors	Business factors
Common to all occupations		Death or disappearance of family members or colleagues Near‐death experience Serious damage to homes Life in evacuation shelters	
Healthcare workers	Single Work and daily life burdens	Death or disappearance of family members Near‐death experience Partial or total destruction of homes Anxiety and fear about radiation exposure	Lack of rest Lack of communication in the workplace
Local government employees	Female Young	Death or disappearance of family members or colleagues Near‐death experience Damage to homes Relocation Life in evacuation shelters	Responding to residents’ complaints Lack of communication in the workplace Lack of rest Disaster‐related work Reassignment
Employees and subcontractors at the Fukushima Daiichi and Fukushima Daini Nuclear Power Plants	Avoidant thinking and resignation coping	Evacuation from tsunami Near‐death experience Significant loss of property	Discrimination and verbal abuse Working immediately after the earthquake Restoration work at the site
Ground self‐defense force personnel			Long‐term dispatch Exposure to dead bodies Living on the same premises as disaster victims
Firefighters		Death or disappearance of family members	Lack of communication in the workplace

### Healthcare workers

The prevalence of PTSS among healthcare workers ranged from 4.6% to 44.4%. The reported risk factors included the death or disappearance of family members, near‐death experiences, complete home destruction, insufficient rest, and poor workplace communication.^15^, ^21^, ^70^ Sato et al.^17^ found that among nurses within 50 km of Fukushima one year after the GEJE, evacuation and difficulty obtaining leave increased PTSS risk. Yamazaki et al.^72^ observed that 2 years after the Niigata‐ken Chuetsu Earthquake, older healthcare and care workers were at higher risk, whereas holding a senior position was protective. Kawashima et al.^22^ reported that two years after the GEJE, disability facility staff with high anxiety regarding future living conditions and those responsible for human resource management were more susceptible. Kobayashi et al.^73^ surveyed hospital staff and municipal public health nurses in Niigata Prefecture after heavy rainfall (2004) and multiple earthquakes (2004, 2007) and identified female sex, total home loss, poor personal or family health, and living in evacuation shelters as risk factors.

The prevalence of psychological distress ranged from 4.0% to 54.4%. Fear and anxiety regarding radiation were repeatedly identified as major contributors.^23^, ^24^ Matsuoka et al. ^23^ reported radiation anxiety as a risk factor for DMAT members one month after the GEJE. Nukui et al.^24^ reported that 4 years after the GEJE, nurses working in hospitals located within a 50–70 km radius of a nuclear power plant had a higher risk of psychological distress if they were single or feared radiation. Sato et al.^17^ identified high resilience and supervisor support as protective factors.

Other studies examined diverse outcomes. Tominaga et al.^18^ found that prior knowledge and skills enhanced empathy and satisfaction and reduced burnout among clinical psychologists deployed after the GEJE. Hatakenaka et al.^28^ observed that DPAT members experienced fluctuating empathy fatigue both during and after deployment. Tsutsui et al.^19^ clarified that grief reactions among hospital staff in tsunami‐affected areas were distinct from PTSS and depression and represented independent symptoms. Kawashima et al.^20^ showed that PDI scores one month post‐GEJE predicted later IES‐R and MBI scores among DMAT members.

In studies involving multiple professions, Sakuma et al.^70^ surveyed public servants in Miyagi Prefecture 1 year after the GEJE and reported a PTSS prevalence of 6.6% among medical personnel, 6.6% among local government employees, and 1.6% among firefighters; the corresponding rates of psychological distress were 14.5%, 14.9%, and 2.6%, respectively. Risk factors included insufficient rest and poor workplace communication among healthcare workers, insufficient rest and disaster‐related duties among local government staff, and poor communication among firefighters.

### Employees and subcontractors at the Fukushima Daiichi and Fukushima Daini nuclear power plants

The reported prevalence of PTSS among nuclear plant employees after the GEJE ranged from 17.2% to 30.6%, with higher rates at Fukushima Daiichi. Common risk factors included discrimination, abuse, and tsunami evacuation.^48^, ^49^, ^51^ The prevalence of psychological distress ranged from 4.0% to 15.8% and was frequently linked to discrimination and verbal abuse.^48^, ^93^ Nagaoka et al.^53^ reported that 36.5% of subcontracted workers met GHQ‐12 ≥ 4, with avoidant or resignation coping associated with greater distress. Hidaka et al.^57^, ^58^ found that decontamination supervisors with higher knowledge of work management reported greater radiation anxiety, whereas on‐site workers experienced anxiety related to isolation, lack of a written contract, and older age.

Ikeda et al.^49^ found that over 40% of Fukushima Daiichi and Daini employees met AIS ≥ 6 within three years post‐disaster. Risk factors for insomnia included discrimination, harassment, major property loss, tsunami experience, evacuation, and witnessing explosions. Discrimination and harassment were associated with all insomnia subtypes (sleep onset, maintenance, and early awakening) and persisted for extended periods.

### Local government employees

A significant number of studies have examined employees in Miyagi and Fukushima Prefectures affected by the GEJE and in Kumamoto Prefecture affected by the Kumamoto Earthquake. The prevalence of PTSS ranged from 6.6% to 35.9%. Common risk factors included resident complaints and criticism, hearing traumatic experiences, poor workplace communication, and home damage.^31^, ^32^, ^33^, ^34^, ^35^ Sakuma et al.^70^ also noted that job reassignment and disaster‐related tasks increased PTSS risk. A lack of perceived control over work tasks,^32^ female sex, and non‐managerial positions^34^ were associated with greater vulnerability.

The prevalence of psychological distress ranged from 4.4% to 17.9%. Reported risk factors included home damage, evacuation, poor communication, excessive workload, and verbal abuse by residents.^31^, ^33^, ^38^, ^39^, ^70^ Gratitude from residents was found to alleviate distress.^32^ Burnout was also observed. Suzuki et al.^41^ reported that 15.9% of officials in Miyagi were at high risk 16 months post‐GEJE. Risk factors included home destruction, health and welfare duties, long working hours, poor communication, evacuation, and handling complaints.

### JGSDF personnel

All studies on JGSDF personnel concerned the GEJE and reported a PTSS prevalence of 0.7%–6.2%. Prolonged deployment, body recovery, and personal disaster exposure were common risk factors.^60^, ^61^ Nagamine et al.^60^ further noted that living near disaster victims increased PTSS risk. The prevalence of psychological distress ranged from 0.5% to 12.8%. Dobashi et al.^59^ found higher distress among personnel in their 20s–30s than among those aged over 50 years. Nagamine et al.^60^ also identified long‐term deployment, exposure to corpses, and cohabitation with victims as risk factors one month post‐disaster.

### Firefighters

All studies on firefighters concerned the GEJE and reported a PTSS prevalence ranging from 0% to 25%. Common risk factors have not been clearly identified. Noda et al.^64^ found that counseling increased avoidance and hyperarousal but reduced intrusion symptoms. PTSS risk was higher among single individuals, those with ≥3 deployments,^65^ and those aged ≥50 years.^66^ Only two studies assessed psychological distress. Yoo et al.^66^ reported a prevalence of 0.9% 1.5 years after the GEJE, whereas Sakuma et al.^70^ reported a prevalence of 2.6% 1 year after the GEJE. Poor workplace communication^70^ was identified as a risk factor. Social support emerged as a protective factor, with colleague support linked to professional growth and family support to gratitude.^66^ In a longitudinal study, Usami et al.^71^ found that the prevalence of PTSS among firefighters declined from 28.8% to 13.5% between seven months and one year after the GEJE, although rates remained higher than those among the Coast Guard Special Rescue Team (6.3%–0%) and rescue divers (20%–0%).

### Police officers

Kamijo et al.^67^ reported PTSS prevalence rates of 25.8% (mild) and 0.9% (moderate) among police officers after the Mt. Ontake eruption. Risk factors included female sex, coping through alcohol or smoking, working ≥7 consecutive days, and supporting victims’ families.

## DISCUSSION

The existing literature primarily comprises cross‐sectional studies, with most disasters analyzed being the GEJE. Participants were predominantly medical professionals, local government employees, and nuclear power plant workers. Reported mental health problems among disaster response workers included PTSS and psychological distress; however, the range was not limited to these outcomes. Other reported conditions included burnout syndrome, sleep disorders, and grief. Few studies have examined flood disasters, volcanic eruptions, or police officers, and these areas remain insufficiently investigated. The following sections examine prevalence rates, common factors contributing to mental health deterioration, and occupation‐specific factors.

### Prevalence of mental health problems

Direct comparison of PTSS and psychological distress prevalence was difficult because of differences in disaster characteristics, study population composition, geographic location, survey timing, and assessment scales across studies. Variation in cutoff values likely contributed to the wide range of reported prevalence estimates. Overall trends indicated relatively high prevalence rates among nuclear power plant workers, healthcare professionals, and local government employees. Initial surveys conducted in Fukushima and Miyagi Prefectures, which were most affected by the nuclear disaster and tsunami, reported comparatively high PTSS prevalence rates.

Among the PTSS prevalence rates reported in the reviewed literature, those for healthcare workers and nuclear power plant employees were as high as those for residents in disaster‐affected areas.^94^, ^95^ Firefighters, JGSDF personnel, and Coast Guard officers exhibited relatively lower rates, which may be partly attributable to prior training and education,^96^, ^97^ although these rates remained higher than those observed in the general Japanese population.^98^ No studies have explicitly demonstrated whether disaster relief activities undertaken by firefighters, JGSDF personnel, and other responders are shorter in duration than those of local government employees or healthcare workers, nor whether such differences contribute to better mental health outcomes. In contrast, a study of JGSDF personnel following the GEJE reported an association between longer deployment periods and PTSD symptoms.^61^ These findings suggest that shorter deployment periods may limit cumulative exposure to disaster‐related stressors and may be associated with better mental health outcomes.

### Factors common across occupations contributing to mental health deterioration

This review identified experiences of mortal danger and the death or disappearance of family members as common risk factors for PTSS across multiple studies. As demonstrated in prior research,^99^, ^100^ a clear correlation exists between the level of exposure to traumatic events and the manifestation of PTSS symptoms. Greater exposure was associated with more pronounced subsequent symptoms. Severe home damage, residence in evacuation centers, long working hours, lack of rest, and poor workplace communication were also identified as contributing factors to the exacerbation of PTSS and psychological distress across various occupations. However, in many studies, “working hours” were not clearly defined, and it remains unclear whether this refers to daily working hours or cumulative duration of disaster‐related service. In disaster response settings, prolonged engagement over extended periods may be more relevant to mental health outcomes. Overall, the deterioration of mental health among disaster responders appears to be largely explained, irrespective of occupation, by the severity of traumatic experiences, workload burden, and changes in living conditions.

### Factors specific to occupation

#### Healthcare workers

Among healthcare workers, the dual role of being both disaster responders and disaster victims appears to exert a distinctive influence on mental health deterioration. Local hospital staff tended to experience higher levels of PTSS and psychological distress than dispatched medical personnel. Local staff may have faced moral dilemmas between professional obligations and personal responsibility to protect their families and themselves. Such dilemmas may contribute to moral injury, to which healthcare professionals are vulnerable, and may exacerbate mental health problems, particularly depressive symptoms. Unlike dispatched personnel, local staff were often unable to avoid stressful duties and were required to continue disaster‐related work over extended periods, which may have resulted in cumulative psychological burden. Anxiety and fear regarding radiation exposure were also specific contributors to psychological distress across multiple studies.^23^, ^24^ Even at designated nuclear disaster hospitals, more than half of nurses expressed concern regarding delayed and genetic effects of low‐dose exposure,^101^ underscoring the need for radiation education for healthcare personnel.

#### Employees and subcontractors at the Fukushima Daiichi and Fukushima Daini nuclear power plants

Nuclear power plant workers were in a distinctive position in that, while serving as disaster responders and being disaster victims themselves, they were also perceived as responsible for the accident. Although they experienced immense suffering as disaster victims, the nuclear accident was regarded as a scandal involving the power company, resulting in defamatory remarks and public criticism directed toward them. Experiences of discrimination and defamation had prolonged effects on PTSD and psychological distress compared with life‐threatening experiences such as tsunami evacuation or major property loss and were also associated with sleep disorders.^48^, ^49^ Findings indicating sustained psychiatric effects among nuclear plant workers are consistent with previous studies of the Chernobyl nuclear power plant accident.^102^, ^103^

#### Local government employees

Responding to residents’ harassment and intense complaints was consistently reported as a distinctive aggravating factor for PTSS and psychological distress among municipal employees. In earthquake‐related tasks such as managing evacuation centers and issuing disaster certificates, encountering intense complaints and anger from residents and hearing their traumatic experiences were common risk factors. Conversely, positive reactions from residents, such as encouragement and expressions of sympathy, were noted as potentially alleviating stress.^32^ Previous reviews have indicated that prioritizing duties over private life, driven by a sense of responsibility as civil servants, is a major stressor.^104^ The Japanese public service system may represent a unique contextual factor. Public employees in Japan are often employed under a lifetime employment system and hold positions of authority while remaining highly susceptible to public criticism, particularly during disaster response and recovery. This combination of long‐term institutional commitment and social scrutiny may contribute to sustained psychological pressure and may partly explain prolonged and unavoidable engagement in disaster‐related duties. Local government employees are often assigned to disaster‐related duties outside their areas of expertise, with limited opportunity for prior training or education.^70^ They are frequently required to remain engaged in disaster response and recovery activities over extended periods, and this prolonged commitment may represent a major source of psychological stress. It may be hypothesized that local government employees face harsher social judgment than specialized disaster response personnel because of their less clearly defined status as responders and lower public recognition.

#### JGSDF personnel

Among JGSDF personnel, in addition to the high intensity of duties involving critical incident stress, close living proximity to disaster victims appears to be associated with exacerbation of PTSS and psychological distress. Tasks involving deceased bodies, such as recovery, transport, and mortuary management, constitute some of the most demanding duties within disaster relief operations and inflict intense catastrophic stress.^105^, ^106^ In this review, multiple studies focusing on JGSDF members consistently reported that duties related to handling human remains were aggravating factors for PTSS and psychological distress.^59^, ^62^ Furthermore, living in close proximity to disaster victims makes it difficult to maintain emotional distance because of excessive empathy or identification, posing a risk for PTSD and psychological distress.^60^ Conversely, ensuring privacy, adequate rest and recreation, and information conversation among personnel may reduce stress.^107^

#### Firefighters

Previous research has reported that emergency responders with longer job tenure are at increased risk of developing psychiatric and post‐traumatic distress.^108^ In this review, several studies identified unmarried status^65^ and older age^66^ as aggravating factors for PTSS among firefighters. In contrast, a previous review^2^ reported mixed findings, with some studies suggesting that longer years of service function as protective factors against mental disorders and PTSS, whereas others reported increased risks or no significant associations. Such inconsistencies may reflect the possibility that longer professional experience entails repeated exposure to traumatic events, leading to symptom recurrence and cumulative effects. Age and length of professional experience may therefore have both protective and aggravating effects on mental health.

#### Police officers

Disaster‐related critical incident stress interventions for police officers have been systematically implemented since the GEJE.^109^ However, empirical evidence remains limited, and in this review only one cross‐sectional study, focused on the Mount Ontake eruption, reported the prevalence of PTSS among police officers.^67^ Previous literature^109^, ^110^ has identified exposure to traumatic events, the death of colleagues or subordinates, bereavement support, and inadequate social support as risk factors for disaster‐related stress, with bereavement support consistent with the findings of this review. Conversely, good communication with senior officers, social support from colleagues and family, and self‐esteem were identified as mitigating factors. These findings underscore the importance of organizational support systems, including leadership support from senior police officers.

### Significance of this study

This study represents the first comprehensive examination of the mental health of disaster relief workers in Japan over the past 30 years. It revealed that PTSS and psychological distress are the primary mental health issues affecting disaster relief workers, with notable differences in their prevalence across occupational groups. Diverse risk factors were identified, and several were consistently observed regardless of occupation.

The findings provide valuable information for organizational managers and administrative agencies when planning, designing, and implementing psychological support measures, including pre‐disaster education and training, psychological counseling, and other interventions aimed at enhancing stress management capacity and psychological resilience among disaster response personnel. These findings may also assist in identifying high‐risk individuals and monitoring their mental health over time.

## LIMITATIONS

The present study has several limitations. First, the literature search was based on a limited number of databases and search terms. Because the research has focused particularly on the GEJE, other disease types may not have been comprehensively covered.

Second, restricting the search to academic journals excluded descriptive studies, non‐English and non‐Japanese papers, review articles, conference abstracts, case reports, activity reports, and intervention studies, potentially introducing publication and selection bias.

Third, as most studies employed cross‐sectional designs, causal relationships cannot be established.

Fourth, many studies adopted retrospective designs, meaning that pre‐disaster risk factors were measured after the disaster.

Fifth, assessment of PTSS and psychological distress relied on self‐administered questionnaires, which do not determine clinical severity. Some disaster responders may avoid seeking support because of stigma surrounding mental illness,^111^ and prevalence rates derived from workplace surveys may not accurately reflect the true situation.

Sixth, many studies targeted specific occupations, and cross‐occupational research remains limited.

## CONCLUSIONS

The primary mental health issues among Japanese disaster responders were PTSS and psychological distress. The prevalence of these conditions and the characteristics of aggravating factors varied by occupation. Trends in prevalence and insight into the stressors identified in this study should inform training and preparedness measures during peacetime, including identification and support of high‐risk individuals.

## AUTHOR CONTRIBUTIONS

Yukari Ito and Hirokazu Tachikawa designed the study. Yukari Ito conducted the primary literature search, and all authors analyzed the data. Yukari Ito created the figures and tables and wrote the first draft of the manuscript. Hirokazu Tachikawa played a major role in revising and editing the draft. Sho Takahashi and Hirokazu Tachikawa amended or added text to the draft. All authors contributed to and approved the final manuscript.

## CONFLICT OF INTEREST STATEMENT

The authors declare no conflicts of interest.

## ETHICS APPROVAL STATEMENT

Ethical approval was not required because only previously published literature was analyzed.

## PATIENT CONSENT STATEMENT

Patient consent was not required because no individual participant data were involved.

## CLINICAL TRIAL REGISTRATION

N/A.

## Data Availability

The data that support the findings of this study are available from the corresponding author upon reasonable request.
